# Profile of violence against the older adults in Brazil according to Brazilian capitals

**DOI:** 10.15649/cuidarte.3233

**Published:** 2024-05-27

**Authors:** Larissa Cipriano Bovolenta, Julia de Lima Mantovani, Fernanda Menegatti Frisanco, Akeisa Dieli Ribeiro Dalla Vechia

**Affiliations:** 1 Faculdade Municipal Professor Franco Montoro, Mogi Guaçu, Brasil. larissabovolenta@gmail.com Faculdade Municipal Professor Franco Montoro Faculdade Municipal Professor Franco Montoro Mogi Guaçu Brazil larissabovolenta@gmail.com; 2 Faculdade Municipal Professor Franco Montoro, Mogi Guaçu, Brasil. juliadelimamantovani@gmail.com Faculdade Municipal Professor Franco Montoro Faculdade Municipal Professor Franco Montoro Mogi Guaçu Brazil juliadelimamantovani@gmail.com; 3 Faculdade Municipal Professor Franco Montoro, Mogi Guaçu, Brasil. fernanda.frisanco@francomontoro.com.br Faculdade Municipal Professor Franco Montoro Faculdade Municipal Professor Franco Montoro Mogi Guaçu Brazil fernanda.frisanco@francomontoro.com.br; 4 Universidade do Estado de Mato Grosso (UNEMAT) campus universitário Professor Eugênio Carlos Stieler, Tangará da Serra, Brasil. akeisa drdv@hotmail.com Universidade do Estado de Mato Grosso Universidade do Estado de Mato Grosso (UNEMAT) campus universitário Professor Eugênio Carlos Stieler Tangará da Serra Brazil akeisa drdv@hotmail.com

**Keywords:** Elderly, Violence, Elder Abuse, Ancianos, Violencia, Abuso de Ancianos, Idosos, Violência, Abuso de Idosos

## Abstract

**Introduction:**

Violence against older adults is a public health problem, being camouflaged in society due to the relationship between aggressors and victims.

**Objective:**

to analyze the profile of violence against older person in Brazil according to data from Brazilian capitals between 2011 and 2019, emphasizing the characteristics of the victims, the aggressors and the violence.

**Materials and Methods:**

This is a temporal ecological epidemiological research, collecting information from the DATASUS database of the Ministry of Health, and consulting the Notifiable Diseases Information System (SINAN). The population was made up of older adults with reported cases of violence between 2011 and 2019.

**Results:**

The majority of cases were female, with schooling corresponding to incomplete 1st-4th grade, white, with physical violence being the most recurrent, with repetition, within the residence, and the main aggressors were the children.

**Discussion:**

The predominance of females is justified by gender/ sociocultural, the majority of victims are white, consistent with Brazilian self declaration, level of education and greater distribution of uneducated older adults. Physical violence is the most prevalent, as it is more visible, favoring its identification. Older adults spend more time at home, triggering risk factors related to the aggressors, with children being more prevalent due to family structural changes.

**Conclusion:**

The study made it possible to profile violence against the old person, exposing the characteristics of this population and identifying possible risk/protective factors; the study also made it possible to identify the importance of correctly filling out the report form and the need to readjust the physical form and the information system.

## Resumen

**Introducción:**

La violencia contra las personas mayores es un problema de salud pública, camuflándose en la sociedad debido a la relación entre agresores y víctimas.

**Objetivo:**

Analizar el perfil de la violencia contra las personas mayores en Brasil según datos de las capitales brasileñas entre 2011 y 2019, destacando las características de la víctima, el agresor y la violencia.

**Materiales y Métodos:**

Se trata de una investigación epidemiológica ecológica temporal, recogiendo información de la base de datos DATASUS del Ministerio de Salud, consultando el Sistema de Información de Enfermedades De Notificación (SINAN). La población estuvo constituida por personas mayores con casos de violencia reportados entre 2011-2019.

**Resultados:**

La mayoría de los casos fueron del sexo femenino, con escolaridad de 1° a 4° grado incompleta, de raza blanca, siendo la violencia física la más recurrente, con repetición, dentro de la residencia y los principales agresores fueron los hijos.

**Discusión:**

El predominio del sexo femenino se justifica por el género/sociocultural, la mayoría de las víctimas son blancas, en consonancia con la autodeclaración brasileña, el nivel de escolaridad y la mayor distribución de ancianos sin escolarización. La violencia física es la más prevalente, ya que es más visible, favoreciendo su identificación. Las personas mayores pasan más tiempo en casa, lo que desencadena factores de riesgo relacionados con el agresor, siendo más prevalentes los hijos debido a los cambios estructurales familiares.

**Conclusión:**

El estudio permitió perfilar la violencia contra el anciano, exponiendo las características de esta población e identificando posibles factores de riesgo/protección; permitió identificar la importancia del correcto llenado del formulario de notificación y la necesidad de reajustar el formulario físico y el sistema de información.

## Resumo

**Introdução:**

A violência contra a pessoa idosa é um problema de saúde pública, sendo camuflada na sociedade devida relação entre agressores e vítimas.

**Objetivo:**

analisar o perfil da violência contra o idoso no Brasil de acordo com dados das capitais brasileiras entre 2011- 2019, enfatizando as características da vítima, do agressor e da violência.

**Materiais e Métodos:**

trata-se de uma pesquisa epidemiológica ecológica temporal, com coleta de informações na base de dados do DATASUS do Ministério da Saúde, consultando-se o Sistema de Informação de Agravos de Notificação (SINAN). A população foi composta por idosos com casos de violência notificados entre 2011-2019.

**Resultados:**

a maioria dos casos foi do sexo feminino, escolaridade de 1^a^-4^a^ séries incompletas, cor branca, sendo a violência física mais recorrente, com repetição, dentro da residência e como principal agressor os filhos.

**Discussão:**

a predominância do sexo feminino se justifica pelo gênero/socioculturais, a maioria das vítimas são brancas, condizendo com a autodeclaração brasileira, nível de escolaridade e maior distribuição de idosos sem instrução. A violência física é a mais predominante, por ser mais visível, o favorecendo sua identificação. O idoso passa mais tempo em casa, desencadeando fatores de risco relativos ao agressor, sendo os filhos mais prevalentes pelas mudanças estruturais familiares.

**Conclusão:**

O estudo possibilitou perfilar a violência contra o idoso, expondo as características dessa população e identificando possíveis fatores de risco/proteção; possibilitou identificar a importância do preenchimento correto da ficha de notificação e a necessidade de readequação da ficha física e do sistema de informação.

## Introduction

Population aging has become an achievement, occurring especially due to reductions in fertility rates and increased life expectancy^1^. According to projections, the world's older adults population will increase between 2015 and 2050 from 12% to 22%, corresponding to 2 billion individuals^2^. At a national level, according to the Brazilian Institute of Geography and Statistics (IBGE), between 2015 and 2050 the older adults population will be 66,265,645, with an aging rate from 37% to 142%^3^.

As aging is a dynamic process, there are several physiological and pathological changes, such as decreased visual and hearing acuity, difficulty in locomotion, heart and respiratory diseases, among others. These factors predispose the older adults to changes in mental and physical capabilities and functionalities, increasing the chance of vulnerabilities, such as violence^4^^-^^5^.

Violence includes embarrassment, use of physical superiority, power struggles, aggression, intra-family and community, economic and psychological abuse, among others. Such facts can lead to financial, mental and emotional losses for the victim and their family, health system expenses, reduced quality of life and death^6^.

Violence against the older adults, according to the World Health Organization, can be defined as any action, single or repeated, or even omission, in a relationship with an expectation of trust, causing harm or distress to the older adults. The literature points to six types of violence against older adults: physical, sexual, psychological, financial/economic, institutional, abandonment and neglect^7^.

According to the WHO, one in every six older adults in the world has experienced violence^5^. In Brazil, in 2019, violence against the older person was the second most reported cause of rights violations, around 48,446 cases (30%)^8^; it is estimated that 5% to 10% of the older population suffers violence^8^^,^^9^.

According to data from DATASUS in Brazil between the years 2011 - 2019, the region that achieved the highest rate of violence against the older adults was the Southeast with 10,874 cases, while the Northeast region had a milestone of 5,431 cases, ranking second and third place we have the central west region with 5,218 cases, in the south region 3,562 cases were recorded, and the last place is the north region with 1,084 cases.

Despite being a public health problem, violence against the elderly is still camouflaged due to the proximity of the victim to the aggressor, such as family members or health workers^8^, with 90% of the incidents occurring in the older adults’ home, 51% of the aggressors being their children and 69% occurring daily, signaling rights violations^5^^,^^8^^,^^9^.

The older adults most vulnerable to violence are women^10^, aged between 76 and 80 years^8^, with financial and physical dependence. Furthermore, they have health problems, such as mental illnesses, low education, live with younger people and are socially isolated^11^^,^^12^.

The importance of early report of cases of violence by the health service is noted to also identify risk factors and take appropriate measures in the face of suspected and confirmed cases; in addition to raising awareness among the population^13^.

The profile identification and factors associated with this phenomenon in the older population enables appropriate health actions for prevention and control. There is still little research on the prevalence of violence against the older adults in Brazilian capitals. Therefore, the question arises: What are the characteristics of violence against older adults in Brazil according to the capitals of Brazil? It is believed that violence against the older person in our country is prevalent, more frequent in older adults with physical, economic and social vulnerabilities.

Therefore, the present work aims to analyze the profile of violence against the older adults in Brazil according to data from Brazilian capitals in the period between 2011 and 2019, emphasizing the characteristics of the victims, the aggressors and the violence.

## Materials and Methods

This is a descriptive ecological epidemiological study. Information was collected about violence against the older adults in Brazilian capitals, totaling data from 26 (twenty-six) capitals and 1 (one) Federal District; the information was obtained from the Ministry of Health's DATASUS database, an online health information system consulted on the website: http://www.datasus.gov.br^14^. The population was made up of older adults with reported cases of violence (confirmed or suspected) between 2011 and 2019. The choice of beginning in 2011 was due to the landmark of violence as a compulsory report problem and the deadline of 2019 was the last year in which the data were updated in the Disease Report Information System (SINAN), within DATASUS, until the data collect. The dataset was stored in the Dryad Digital Repository DataSet^15^.

### Inclusion criteria

Be 60 years of age or older, with a case of violence reported (suspected or confirmed) by a professional in the Unified Health System (SUS) between 2011 and 2019, first through the physical report form (paper) and, subsequently, transferred to the online information system. Only data on interpersonal violence and by Brazilian capital were used.

### Exclusion criteria

Data from the subcategories called “ignored” and “blank” were excluded, as such data, even with the largest quantities, did not corroborate the obtaining of data that qualified the real profile of violence.

### Data collection and analysis

The information was taken from DATASUS, using the Notifiable Diseases Information System (SINAN) as a source. It appears that the collection was carried out using the values of Brazilian capitals as representatives of the values of violence against the older adults by states. Data were collected related to the number of cases per capital, the number of cases per year through the sum of cases in the capitals, the characteristics of the aggressor (life cycle and relationship with the victim), the characteristics of the victim (sociodemographic variables) and the characteristics of the violence (place of occurrence, repetition, type, suspected alcohol use). Subsequently, the results were described in terms of the observed characteristics and justified according to current literature (studies between 2017 and 2021). The data obtained were transcribed into the Microsoft Office Excel software statistical package, version 2014, for data processing and presentation in graphs and tables to better describe the results, grouped, systematized and analyzed. The database used for storage was Microsoft 365, version 2020.

### Ethical aspects

This research was based on public domain data, made available by the Ministry of Health and which protects the identity of the subjects

## Results

It is observed that in population terms, during the period studied, there was a higher prevalence offemale victims (57.30%), white color/race (47.32%). As for the victims' education, the main level was incomplete 1st to 4th grade (30.02%), according to Table 1.

**Table 1:** Characteristics of older adults who are victims of violence, according to sociodemographic variables, according to Brazilian capitals, 2011-2019

Variables	n	%
Sex		
Male	11.205	42.82
Female	14.960	57.30
Color/Race		
White	10.681	47.32
Black	2.550	11.30
Yellow	357	1.58
Brown	8.892	39.39
Indigenous	92	0.41
Schooling		
Illiterate	1.550	12.52
Incomplete 1st to 4th grade	3.716	30.02
Complete 4th grade	1.271	10.27
Incomplete 5th to 8th grade	1.816	14.67
Complete elementary school	1.307	10.56
Incomplete high school	541	4.37
Complete high school	1.432	11.57
Incomplete higher education	166	1.34
Complete higher education	580	4.69

Source: Ministry of Health/ SVS - Notifiable Diseases Information System - Sinan Net.

The main type of violence was physical (41.92%), the place of greatest occurrence was at home (77.32%), violence was repeated in 63.80% of cases and in the majority (66.87%) there was no suspected of alcohol consumption (Table 2).

**Table 2:** Characteristics of violence against older adults, according to variables of type, location, repetition and use of alcohol, according to Brazilian capitals, 2011-2019

Variables	n	%
Type of Violence		
Physical	14.462	41.92
Psychological	6.063	17.57
Torture	356	1.03
Financial	2.138	6.20
Human trafficking	10	0.03
Sexual	695	2.01
Negligence/Abandonment	10.414	30.19
Legal intervention	54	0.16
Others	307	0.89
Place of occurrence of violence		
Residence	17.738	77.32
Collective housing	328	1.43
School	29	0.13
Sports practice place	15	0.07
Bar or similar	263	1.15
Public highway	2.681	11.69
Commerce and services	707	3.08
Industry/construction	15	0.07
Others	1.166	5.08
Repeated violence		
Yes	10.885	63.80
No	6.175	36.20
Suspected alcohol use		
Yes	4.780	33.13
No	9.649	66.87

Source: Ministry of Health/SVS - Notifiable Diseases Information System - Sinan Net

In Table 3 we present the main characteristics of the aggressor of violence against the older person. The main relationship between aggressor-victim was that of son/daughter (41.55%), and in relation to the life cycle of this aggressor, the main one was the adult (70.32%).

**Table 3:** Characteristics of the aggressor of violence against the older person, according to relationship variables with the victim and the aggressor's life cycle, according to Brazilian capitals, 2011-2019

Variables	n	%
Relationship with the victim		
Spouse	2.263	9.23
Ex-spouse	418	1.70
Boyfriend/girlfriend	108	0.44
Ex-boyfriend/ex-girlfriend	76	0.31
Son/daughter	10.191	41.55
Brother	1.147	4.68
Friend/acquaintance	2.097	8.55
Unknown	2.954	12.04
Caregiver	801	3.27
Employer/chief	35	0.14
Person with institutional relationship	292	1.19
Police/law enforcement officer	63	0.26
Other ties	4.081	16.64
Aggressor life cycle		
Adolescent	392	3.60
Young people	1.071	9.84
Adults	7.654	70.32
Older adults	1.767	16.23

Source: Ministry of Health/SVS - Notifiable Diseases Information System - Sinan Net.

**Figure 1:**
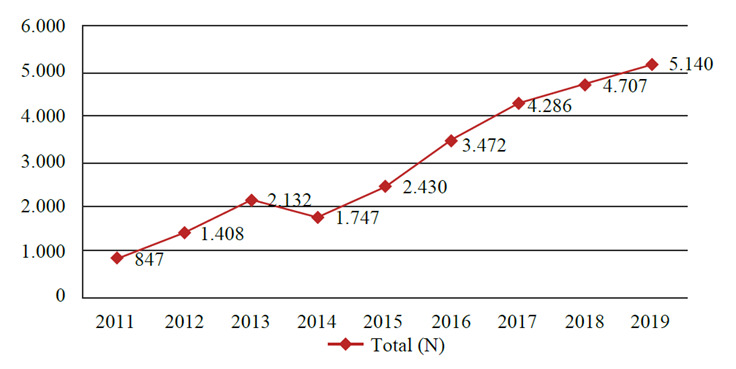
Number of reports of violence against older adults per year, according to the sum of values for Brazilian capitals, 2011-2019

Source: Ministry of Health/ SVS - Notifiable Diseases Information System - Sinan Net

**Figure 2:**
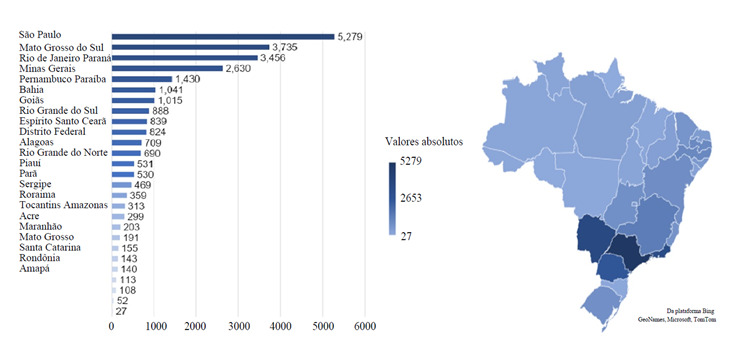
Number of reports of interpersonal violence against older adults by Brazilian capital as representatives of Brazilian states, 2011-2019

Source: Ministry of Health/ SVS - Notifiable Diseases Information System - Sinan Net

It is observed that in the prevalence of violence per year according to the capitals of Brazil, in 2019 there were more reports, 5.140 cases, followed by 2018 with 4.707, and 2017 with 4.286, as shown in Figure 1. Figure 2 shows the capitals of Brazil, represented by the name of their states, with the absolute frequencies of interpersonal violence. The highest values obtained were in São Paulo (SP) with 5.279 reports, followed by Campo Grande (MS) with 3.735 and Rio de Janeiro (RJ) with 3.456. The capitals with the lowest reported values were Macapá (AP) with 27, Porto Velho (RO) with 52 and Florianópolis (SC) with 108 reported cases.

## Discussion

The research identified a higher prevalence of female victims, white color/race, with incomplete 1st to 4th grade education. The main type of violence was physical, the place of greatest occurrence being the residence, indicated with repeated episodes and unrelated to alcohol consumption. Regarding the aggressor, the main one was the son/daughter, also showing a high prevalence of unknown aggressors; Furthermore, the aggressors were adults. Regarding reports per year in the capitals, 2019 had more cases, with the capital São Paulo having the highest values.

In this study, there was a prevalence of violence against older women, which may be related to gender/ sociocultural issues^16^. The literature reports a correlation between violence against the older adults and the feminization of old age, that is, there are older women than older men, explained by differences in life expectancy between the sexes^17^^,^^18^. Despite their longevity, women have more comorbidities, lower quality of life and greater dependence on caregivers, increasing the chance of violence^18^^,^^19^. Thus, although women live longer, they become more prone to abuse, contributing to the sociocultural aspects of gender and aging^16^^,^^19^.

Regarding the race of the victims, the study characterized a higher prevalence of the white race, probably related to the characteristics of the Brazilian population, the majority of which self-declare as white, 42.7% of the population, 45.1% of mixed race and 8.9% of black. Furthermore, this issue may be related to the process of inequality in access to information and studies for the black population^20^.

Regarding education, there is a prevalence of incomplete grades 1st to 4th; however, there is no agreement in the literature between this level of education and violence against the older adults^13^. In Brazil, there is a greater distribution of older adults with no education or with incomplete elementary education, which could justify^21^. Even without consensus, literature justifies education in terms of older adults’ perception of violence suffered, that is: the higher the education, the lower the prevalence of violence^22^^,^^25^. According to the literature, it is possible to correlate low education with dependence in activities of daily living, a risk factor for abuse^20^. There are also studies that link low education with income: the lower the education, the lower the income and the more prone to violence^12^^,^^24^.

Regarding the type of violence, physical violence stands out, which can be justified because it is more noticeable, that is, as it causes visible injuries, such as bruises, it becomes easier for another person to identify it^26^^,^^27^.

The study presented residence as the place of greatest occurrence, which is consistent with national literature. The older person, due to the aging process, such as dependence on certain activities, tend to stay at home longer, configuring a risk factor^21^^,^^27^^,^^29^. Furthermore, it is possible to correlate the higher prevalence of cases involving older women and the cases of abuse occurring at home, since in the national culture, women spend more time at home, with less external contact^23^^,^^30^.

Repeated violence, listed as prevalent, is not explained in the literature, but studies assume explanations, such as failure to report the aggressor - who in most cases is known - due to fear of loss of connection, fear, shame, etc., causing the violence to continue^31^^,^^33^. In this way, whoever identifies the abuse becomes, in most cases, an outsider, especially because the older person seeks to justify the abuse suffered and does not identify it as violence^31^. Due to the proximity to their aggressor being linked to repetition, many older adults are unable to perceive the violence^16^^,^^27^^,^^31^. Thus, repetition demonstrates that behind each reported case of violence, there were several unreported episodes^33^^,^^34^.

Regarding the aggressor, it is observed that children are the most prevalent in this study, as in the literature. This can be justified by changes in family structures, such as children separating from their spouses, lack of economic stability, among others^16^^,^^27^. There is, therefore, a need for family reorganization, as the child will take care of the older person (spontaneous or imposed)^10^^,^^19^^,^^35^

Although the children become the caregivers, they often do so informally and empirically, not recognizing the aging process and making caring tiring and stressful, which can create a risk of violence^10^^,^^16^^,^^35^^,^^37^.

Regarding the prevalence of unknown aggressors, a finding of this study, violence can also be related to ageism, that is, prejudice due to age^38^^,^^39^. The existing prejudice against older adults can be evidenced by the way society looks at the older adults, for example, not recognizing the loss of certain skills and lack of knowledge about senescence and vulnerabilities^16^^,^^22^^,^^35^^,^^37^^,^^40^^,^^41^.

The data presented in “other ties” were not described in the form provided by SINAN, as they include other data than those already specified in the table. This is justified by underreporting and inadequate completion of forms at the time of report, which is a reality still present today, already covered in studies, it is estimated that there are five omitted or inconclusive cases for each reported^16^.

The data collected, based on chronology, tends to increase the number of cases over the years; however, there is still a consensus in the literature. However, some hypotheses, such as the fact that the issue of violence is more discussed and publicized, that is, dissemination of the rights of the older adults and the way to recognize when these rights are disrespected^32^.

Thus, as society begins to have more knowledge about the subject, there is greater encouragement to report cases (suspected or confirmed) of violence to the authorities, as well as more intense struggles to reduce the number of events^23^^,^^42^. In this way, it is possible to provide more training to health professionals, who can detect cases early, as well as readapting reporting tools^23^^,^^43^^,^^44^.

It is observed that the State of São Paulo has the highest number of reports; however, there is no agreement in the literature. Even so, it is possible to point out that the State of São Paulo, due to its development, tends to have a structured support network^32^^,^^33^^,^^44^. It is also observed how local political structures can influence the information that is transmitted, that is, São Paulo publicizes cases more, allowing greater knowledge of the problem and the adoption of resolving measures^32^^,^^42^^,^^43^.

During the data collection, there was a lot of underreporting and filling failures in the SINAN database, which may occur due to differences between the physical form (paper) and the system form; that is, data filled in on the SINAN physical form is not included in the system, such as the presence of deficiencies or disorders, a factor that could help in understanding risk or protective factors.

The fact that underreporting is still observed means that the fight for the rights of the older person is hampered, as it hinders the action of public policies, as the amount of reliable data is scarce^32^. Underreporting can occur due to lack of preparation and fear, as well as professionals' lack of knowledge about filling out the form and even its importance for recording and using this data^31^^,^^34^^,^^45^.

For nursing professionals, approaching the victim involves a complexity of factors, not analyzing the case in isolation, but the entire life context, as all factors can become a risk or protection for violence^31^^,^^45^. However, according to the literature, there is a lack of preparation among professionals regarding the identification of cases, how to proceed in confirmed or suspected cases and the fear of involvement with the aggressors. Another factor is the difficulty in referring/counter-referring, which may be related to the undergraduate degree of this professional^31^^,^^45^.

Nurses often feel afraid and choose to report only on Dial 100, which directly interferes with SINAN^31^^)^ reports. Therefore, it is important to train professionals and provide support to the health network^45^^,^^46^.

Finally, it is worth highlighting the importance of the Family Health Strategy (FHS) as with the multidisciplinary team it becomes easier to identify and report mistreatment. In this sense, it is possible to observe that the older adults’ registration in the FHS and the bond with the team becomes a protective factor, as in addition to having the monitoring of the nurses within the BHU, they are also able to make home visits that can report violence^13^^,^^36^.

In view of this, this study correlates with the practice to expand knowledge regarding the profile of violence against the older adults, allows new tools for measuring cases and greater investments in expanding and disseminating the service sectors and reports ofviolence against the older adults, proposing intervention measures to minimize damage.

## Conclusion

The present study allowed us to characterize violence against the older adults in Brazil as more prevalent among females, white people and those with incomplete elementary education, with physical violence being the most recurrent, with repeated episodes, occurring within the residence and children being the aggressors. Although not justifiable, children as the main aggressors may be related to a lack of preparation, as well as the replication of aggressions suffered.

Inadequate completion of report forms results in data not being solid, indicating the uncertainty of the problem. It is also possible to consider that professionals have difficulties filling out the form or the importance of the data, fear, lack of preparation or, even if unjustifiable, excessive daily activities; Regardless, training must take place.

Nurses can notify cases; Those who work at the BHU have greater bonds with the patients, giving this population the opportunity to feel confident in talking about abuse. In other words, these professionals are capable of identifying violence and intervening.

## References

[1] Martin Dantas EH, Souza Santos CA (2017). Aspectos biopsicossociais do envelhecimento e a prevenção de quedas na terceira idade.

[2] Pan American Health Organization Envelhecimento saudável.

[3] Instituto Brasileiro de Geografia e Estatística (2018). Coordenação de População e Indicadores Sociais. Gerência de estudos e Análises da Dinâmica Demográfica. Projeção da população do Brasil e Unidade da Federação por sexo e idade para o período 2010-2060.

[4] World Health Organization (2015). World report on ageing and health.

[5] World Health Organization (2016). Elder abuse: the health sector role in prevention and response.

[6] Souza Minayo MC (2006). Violência e Saúde.

[7] World Health Organization (2014). The Toronto Declaration: on the Global prevention of elderabuse.

[8] Ministério da mulher (2019). da família e dos direitos humanos. Disque direitos humanos: relatório 2019.

[9] Arantes RC, Nunes MA, Bertóglio M, Clos W, Alves CB, Musial DC, Barroso AES, Marcolino- Galli JF, Rocha F (2020). Políticas sociais e gerontologia: diálogos contemporâneos.

[10] Lopes ED, Ferreira ÁG, Pires CG, Moraes MC, D'Elboux MJ (2018). Elder abuse in Brazil: an integrative review. Rev Bras Geriatr Gerontol.

[11] Secretaria de Direitos Humanos da Presidência da república (2014). Manual de enfrentamento à violência contra a pessoa idosa.

[12] Santos MAB dos, Moreira R da S, Faccio PF, Gomes GC, Silva V de L (2020). Fatores associados à violência contra o idoso: uma revisão sistemática da literatura. Ciênc saúde coletiva.

[13] Alencar FD, Moraes JR (2018). Prevalência e fatores associados à violência contra idosos cometida por pessoas desconhecidas, Brasil, 2013. Epidemiologia Serv Saude.

[14] DATAUS (2007). Ministério da Saúde. Doenças e agravos de notificação.

[15] Bovolenta LC, Mantovani JL, Frisanco FM, Vechia ADRD (2023). Perfil da violência contra o idoso no Brasil segundo as capitais brasileiras. Dryad Digital Repository.

[16] Santos ACP de O, Silva CA da, Carvalho LS, Menezes M do R de (2007). A construção da violência contra idosos. Revbras geriatr gerontol.

[17] Barros RL, Leal MC, Marques AP, Lins ME (2019). Violência doméstica contra idosos assistidos na atenção básica. Saude Em Debate.

[18] Lopes ED, D'Elboux MJ (2021). Violência contra a pessoa idosa no município de Campinas, São Paulo, nos últimos 11 anos: uma análise temporal. Rev Bras Geriatr Gerontol.

[19] Jeon GS, Cho SI, Choi K, Jang KS (2019). Gender Differences in the Prevalence and Correlates of Elder Abuse in a Community-Dwelling Older Population in Korea. Int J Environ Res Public Health.

[20] Instituto Brasileiro de Geografia e Estatística- IBGE (2020). Síntese de Indicadores Sociais: uma análise das condições de vida da população brasileira.

[21] Dias AL, Santos JD, Monteiro GK, Santos RC, Costa GM, Souto RQ (2020). Association of the functional capacity and violence in the elderly community. Rev Bras Enferm.

[22] Alarcon MF, Damaceno DG, Lazarini CA, Braccialli LA, Sponchiado VB, Marin MJ (2019). Violência sobre a pessoa idosa: um estudo documental. Rev Rene.

[23] Brandão BM, Santos RC, Araújo-Monteiro GK, Carneiro AD, Medeiros FD, Souto RQ (2021). Risk of violence and functional capacity of hospitalized elderly: a cross-sectional study. Rev Esc Enferm USP.

[24] Du P, Chen Y (2021). Prevalence of elder abuse and victim-related risk factors during the COVID-19 pandemic in China. BMC Public Health.

[25] Sousa RCRD, Araújo GKND, rQ Souto, Santos RCD, Santos RDC, Almeida LR de (2021). Factors associated with the risk of violence against older adult women: a cross-sectional study. Rev Latino-Am Enfermagem.

[26] Rodrigues RAP, Monteiro EA, Santos AMR dos, Pontes M de L de F, Fhon JRS, Bolina AF (2017). Older adults abuse in three Brazilian cities. Rev Bras Enferm.

[27] Hohendorff JV, Paz AP, Freitas CPP, Lawrenz P, Habigzang LF (2018). Caracterização da violência contra idosos a partir de casos notificados por profissionais da saúde. Rev. SPAGESP.

[28] Maia PHS, Ferreira EF e, Melo EM de, Vargas AMD (2019). Occurrence of violence in the elderly and its associated factors. Rev Bras Enferm.

[29] Matos NM de, Albernaz E de O, Sousa BB de, Braz MC, Vale MS do, Pinheiro HA (2019). Profile of aggressors of older adults receiving care at a geriatrics and gerontology reference center in the Distrito Federal (Federal District), Brazil. Rev Bras Geriatr Gerontol.

[30] Andrade FM, Ribeiro AP, Bernal RT, Machado ÍE, Malta DC (2020). Perfil dos atendimentos por violência contra idosos em serviços de urgência e emergência: análise do VIVA Inquérito 2017. Rev Bras Epidemiología.

[31] Alarcon MFS, Damaceno DG, Cardoso BC, Sponchiado VBY, Braccialli LAD, Marin MJS (2020). Percepção do Idoso acerca da violência vivida. Rev. baiana enferm.

[32] Rocha RC, Côrtes MCJW, Dias EC, Gontijo ED (2018). Violência velada e revelada contra idosos em Minas Gerais-Brasil: análise de denúncias e notificações. Saúde debate.

[33] Rodrigues RAP, Chiaravalloti-Neto F, Fhon JRS, Bolina AF (2021). Spatial analysis of elder abuse in a Brazilian municipality. Rev Bras Enferm.

[34] Barufaldi LA, Souto RM, Correia RS, Montenegro MD, Pinto IV, Silva MM (2017). Violência de gênero: comparação da mortalidade por agressão em mulheres com e sem notificação prévia de violência. Cienc Amp Saude Coletiva.

[35] Winck DR, am Alvarez (2018). Percepção de enfermeiros da estratégia saúde da família acerca das causas da violência contra a pessoa idosa. Rev APS.

[36] Moraes CL, Marques ES, Ribeiro AP, Souza ER (2020). Violência contra idosos durante a pandemia de Covid-19 no Brasil: contribuições para seu enfrentamento. Ciênc saúde coletiva.

[37] Matos NM, Braz MC, Albernaz EO, Sousa BB, Pinheiro HA, Ferreira DTT (2021). Mediação de conflito: soluções propostas em atendimento a casos de violência contra a pessoa idosa. Rev Bras Geriatr Gerontol.

[38] Chang ES, Monin JK, Zelterman D, Levy BR (2021). Impact of structural ageism on greater violence against older persons: a cross-national study of 56 countries. BMJ Open.

[39] Associação Pan Americana de Saúde-OPAS (2021). Relatório mundial sobre o idadismo: resumo executivo. Pan American Health Organization.

[40] Wanderbroocke ACNS, Camargo D, Rossoni A, Schmitte GR, Costa J, Macedo VB (2020). Sentidos da violência psicológica contra idosos: experiências familiares. Pensando famílias.

[41] Ribeiro MNS, Santo FHE, Diniz CX, Araújo KB, Lisboa MGL, Souza CRS (2021). Evidências científicas da prática da violência contra a pessoa idosa: revisão integrativa. Acta paul enferm.

[42] Piuvezam G, Aquino AF, Rocha KP, Oliveira VN, Santos RC, Bezerra IN (2019). Distribuição da morbimortalidade por violência em idosos no Rio Grande do Norte. Av En Enfermeria.

[43] Castro VC, Rissardo LK, Carreira L (2018). Violence against the Brazilian elderlies: an analysis of hospitalizations. Rev Bras Enferm.

[44] Silva GCN, Almeida VL, Brito TRP, Godinho MLSC, Nogueira DA, Chini LT (2018). Violência contra idosos: uma análise documental. Aquichan.

[45] Oliveira KS, Carvalho FP, Oliveira LC, Simpson CA, Silva FT, Martins AG (2018). Violência contra idosos: concepções dos profissionais de enfermagem acerca da detecção e prevenção. Rev Gauch Enferm.

[46] Poltronieri BC, Souza ER de, Ribeiro AP (2019). Violência no cuidado em instituições de longa permanência para idosos no Rio de Janeiro: percepções de gestores e profissionais. Saude soc.

